# Analysis of volatile organic compounds in exhaled breath of blood cancer patients identifies products of lipid peroxidation as biomarkers for lymphoma detection

**DOI:** 10.1002/hem3.70168

**Published:** 2025-07-18

**Authors:** Lotte C. A. Stiekema, Hsuan Chou, Amy Craster, Bela Wrench, Katiuscia Bianchi, Paolo Gallipoli, Jeffrey K. Davies, John G. Gribben, John C. Riches

**Affiliations:** ^1^ Centre for Haemato‐Oncology, Barts Cancer Institute Queen Mary University of London London UK; ^2^ Owlstone Medical Ltd. Cambridge Cambridgeshire UK; ^3^ Centre for Cancer Cell and Molecular Biology, Barts Cancer Institute Queen Mary University of London London UK

Volatile organic compounds (VOCs) are continuously generated by the human body and carry considerable information about an individual's physiological and metabolic status. The field of VOC analysis is rapidly developing, demonstrating that patients with cancer have distinct VOC profiles in their blood, urine, sweat, and breath. Since initial observations in lung cancer, there have been numerous reports of the differential production of VOCs in other solid cancers, including pancreatic, gastric, esophageal, prostate, ovarian, and breast cancer.^1^, ^2^, ^3^, ^4^, ^5^, ^6^ This led to the clinical development of breath analysis as a noninvasive tool for the screening, diagnosis, and monitoring of these cancers. Despite this, there have been no studies using breath VOC analysis in hematological malignancies, despite in vitro observations identifying VOCs that can discriminate acute myeloid leukemia (AML) cells from normal cells.^7^ VOC analysis that specifically identifies blood cancers has great potential for early detection and for monitoring treatment effectiveness, leading to increased survival and reduced healthcare costs.

Here, we report the first study of VOCs in the exhaled breath of patients with hematological cancers. Seventy‐four individuals (46 blood cancer patients, 28 healthy controls) were recruited between August 2020 and March 2022, and their breath was collected using Breath Biopsy® technology developed by Owlstone Medical Ltd. (Cambridge, UK). Individuals had to be ≥18 years old at collection and had to have a diagnosis of acute leukemia or high‐grade lymphoma (blood cancer patients) or be otherwise healthy with no major comorbidities (controls). Inclusion and exclusion criteria are listed in Table S1. The patients were all tested before the start of definitive antineoplastic therapy (within 7 days). The only exception to this was for some patients with acute leukemia who were receiving cytoreductive therapy (e.g., hydroxycarbamide) to control their blast count as required by their clinical status. The aim was to establish whether there was a distinct VOC profile that could distinguish between blood cancer patients and healthy individuals. Breath samples were collected over 8–12 min using a ReCIVA Breath Sampler (Owlstone Medical Ltd.) onto a Breath Biopsy Cartridge consisting of four Tenax TA + Carbograph 5TD sorbent tubes (Markes International, Bridgend, UK) (Supplementary Methods and Figure S1). The ReCIVA Breath Sampler enables monitoring of subjects' breathing in real‐time using pressure sensors, which trigger sampling pumps to collect breath at specific stages of the respiratory cycle. This allows the breath collection to focus on exhaled breath from the lungs and exclude air from the mouth and upper airway (anatomic dead space). The system also excludes end tidal‐breath due to previous observations demonstrating poor reproducibility. Breath samples were curated to confirm acceptable quality before data analysis, with samples deemed to have unacceptable quality (e.g., due to pressure inconsistencies representing potential ReCIVA Breath Sampler leakage during collection) excluded from data analysis. Samples from 27 healthy controls (96% of those sampled) and 36 blood cancer patients (75% of those sampled) were considered of sufficient quality for analysis. The 36 patients with blood cancers included 17 patients with AML, 8 patients with acute lymphoblastic leukemia (ALL), and 11 patients with high‐grade lymphoma; see Table S2 for clinical background information. Samples were analyzed using the OMNI Breath Biopsy® Platform (Owlstone Medical Ltd.). Samples were dry purged in a thermal desorption instrument (TD‐100, Markes International) to remove excess water at receipt, and then stored until all samples had been collected and analyzed as one batch. Samples were thermally desorbed and analyzed by gas chromatography‐mass spectrometry (GC‐MS: QExactive GC Hybrid Quadrupole‐Orbitrap Mass Spectrometer, Thermo Scientific, Waltham, MA, USA). Raw chromatograms were imported into Compound Discoverer version 3.2 (Thermo Scientific) to generate a list of molecular features (MFs). The VOCs were then identified by comparison of the MFs to reference spectra from the National Institute of Standards & Technology (NIST, Gaithersburg, USA) electron impact library and/or Owlstone Medical's internal high‐resolution accurate mass (HRAM) library. In addition to providing identification of the VOCs, the MF identification process also provides a score that reflects the percentage match with the reference spectra.

A total of 394 MFs were included for further analysis; each MF was labeled with a unique numerical identifier composed of retention time and the mass‐per‐charge ratio (*m*/*z*) of the ion used for quantitation. For example, mf_21.259_69.07 is a feature eluting at 21.259 min and quantified using the ion at 69.07 *m*/*z*. Of the features found, 315 MFs (80%) were assigned tentative identifications generated by comparisons to the NIST reference library; 49 MFs (12%) were also assigned an identifier using Owlstone's HRAM library. For the univariate analysis of whether individual VOC abundances differed between two groups, linear regression was used to identify the compounds of interest for each disease‐control comparison. Multiple testing correction using the Benjamini–Hochberg method was applied.

Table 1 lists the top 15 compounds that were differentially present in the breath of patients with high‐grade lymphoma compared to healthy controls. There was variation in the VOC levels with 45% present at higher levels in the breath of patients with high‐grade lymphoma. Notably, four VOCs significantly increased in the breath of high‐grade lymphoma patients were alkanes: 4‐methyldecane, decane, 4‐methylundecane, and 2,3,5‐trimethylhexane—remarkably comparable to compounds previously identified at increased levels in the breath of patients with lung cancer.^5^ Increased levels of methylated alkanes are by‐products of lipid peroxidation and subsequent degradation of long‐chain polyunsaturated fatty acids by reactive oxygen species under oxidative stress conditions.^8^ This is of significant interest as lipid peroxidation and ferroptosis have been implicated in the tumorigenesis, progression, and drug resistance of lymphoma, with several ferroptotic molecules identified as putative biomarkers and therapeutic targets.^9^

**Table 1:** A list of volatile organic compounds (VOCs) that were differentially detected in the breath of patients with high‐grade lymphoma compared to healthy controls.

**High‐grade lymphoma**					
ID	Tentative ID	NIST score	P‐value	FC	ROC AUC
mf_15.572_85.028	5‐Oxotetrahydrofuran‐2‐carboxylic acid	81	0.0002	0.46	0.94
mf_21.089_93.07	Eucalyptol	96	0.002	0.50	0.90
mf_19.635_124.088	2,3‐Dehydro‐1,8‐cineole	96	0.004	0.53	0.88
mf_26.05_108.093	(+)‐2‐Bornanone	96	0.004	0.78	0.88
mf_7.09_68.05	Isobutyronitrile	82	0.007	0.73	0.93
mf_19.745_71.086	Decane, 4‐methyl‐	94	0.009	1.66	0.87
mf_12.62_85.101	Hexane, 2,3,5‐trimethyl‐	91	0.013	1.82	0.85
mf_12.864_74.019	Dihydro‐2(3*H*)‐thiophenone	91	0.013	0.25	0.86
mf_17.921_93.07	Santolina triene	95	0.019	0.47	0.86
mf_11.776_163.875	Tetrachloroethylene	93	0.019	0.17	0.91
mf_20.449_138.14	Cyclohexene, 3‐methyl‐6‐(1‐methylethyl)‐	96	0.020	1.04	0.85
mf_27.083_133.065	Benzaldehyde, 2,4‐dimethyl‐	94	0.021	1.40	0.82
mf_19.583_71.086	Decane	96	0.024	1.89	0.86
mf_22.893_71.086	Undecane, 4‐methyl‐	92	0.036	1.71	0.86
mf_8.379_73.011	Sulfide, allyl methyl	95	0.037	0.11	0.81

*Note*: VOCs that were more abundant in the breath of lymphoma patients are highlighted in orange; those that were more abundant in the breath of healthy controls are highlighted in blue.

Abbreviations: FC, fold change; NIST, National Institute of Standards & Technology; ROC AUC, receiver operating characteristic area under the curve.

Table 2 lists the top 15 compounds that were differentially present in the breath of patients with acute leukemia compared to healthy controls. In contrast to patients with high‐grade lymphoma, comparison of acute leukemia patients versus healthy controls showed approximately 65% of VOCs having negative fold‐changes, that is, a lower abundance of VOCs in the breath of the disease group. Even though this was an untargeted analysis with the potential to identify 10,000s of VOCs, individual VOCs were recurrently identified. For example, methanethiol was found to have significantly lower abundance in the breath of patients with both AML and ALL compared to healthy controls. Methanethiol is a compound that occurs naturally in nuts and cheese and can also be produced from methionine by methionine γ‐lyase, a bacterial enzyme found in, for example, Clostridiales, Pseudomonas.^10^ Similarly, allyl methylsulfide is another dietary VOC, derived from plants of the genus *Allium*, such as garlic and onion, and can also be produced by the gut microbiome.^11^, ^12^ Several other dietary VOCs were identified, including 2,6‐diethyl‐pyrazine and dihydro‐2(3*H*)‐thiophenone found in coffee, and which along with 2‐butyltetrahydrothiophene are also Millard reaction products generated by high‐temperature cooking. In addition, 2,3‐dehydro‐1,8‐cineole is found in tea and is a metabolized product of eucalyptol,^13^ and 3,3‐dimethylhexane can also be found in teas and other herbs and spices. Taken together, the observation that these were all found with decreased abundance in the acute leukemia patients points towards the impact of alterations in diet and/or antimicrobial therapy on the expired breath “volatome.” Notably, virtually all (96%) of the acute leukemia patients were inpatients at the time of sampling with a majority (60%) receiving treatment with broad‐spectrum antibacterials. Clinical studies in pediatric ALL subjects^14^ and AML subjects^15^ have indicated a microbiome diversity decrease and compositional change in these cancers. In contrast, the healthy controls were all members of the healthcare team recruited to meet the ethical requirements of having had a recent negative COVID‐19 test before sampling during the study. Therefore, it is likely that the alterations in diet associated with admission to hospital and the differential use of antibiotics between the healthy controls and acute leukemia groups were confounding factors. Notably, this was less of an issue with the patients with high‐grade lymphoma as the majority (64%) were outpatients at the time of sampling, and likely to be having normal diet without concomitant antibacterial therapy. Although the healthy controls (mean age 37 years) were younger than the patients with high‐grade lymphoma (because of restrictions due to the COVID‐19 pandemic), these patients had a comparable age to the AML cohort (mean age 59.7 years [lymphoma] vs. 56.7 years [AML]; P = 0.93). As the increase in alkanes/methylated alkanes was only seen in patients with high‐grade lymphoma, age is not likely to be accounting for the differences observed.

**Table 2:** A list of volatile organic compounds (VOCs) that were differentially detected in the breath of patients with acute leukemia compared to healthy controls.

Tent ID	Tentative ID	NIST score	P‐value	FC	ROC AUC
*(A) Acute myeloid leukemia*					
mf_14.847_59.037	Acetamide	96	0.003	0.65	0.85
mf_11.776_163.875	Tetrachloroethylene	93	0.007	0.20	0.81
mf_1.757_43.054	Isobutane	80	0.009	0.54	0.84
mf_19.635_124.088	2,3‐Dehydro‐1,8‐cineole	96	0.013	0.74	0.81
mf_19.715_210.991	1,4‐Benzenedicarboxylic acid, bis(2‐hydroxyethyl) ester	55	0.013	0.73	0.81
mf_1.988_48.003	Methanethiol	97	0.015	0.32	0.76
mf_13.457_149.045	1,3‐Dioxolane, 2‐(3‐chloropropyl)‐2‐methyl‐	53	0.020	0.58	0.79
mf_22.752_135.092	Pyrazine, 2,6‐diethyl‐	79	0.030	0.19	0.84
mf_2.476_43.054	Pentane	79	0.031	0.88	0.76
mf_16.793_93.07	Butanoic acid, 3‐methyl‐, 1‐ethenyl‐1,5‐dimethyl‐4‐hexenyl ester	86	0.031	0.60	0.75
mf_9.221_117.037	Acetic acid, TMS derivative	96	0.037	0.56	0.79
mf_5.373_56.062	Cyclopentane, methyl‐	HRAM	0.040	1.19	0.77
*(B) Acute Lymphoblastic Leukemia*					
mf_22.258_106.065	4‐Cyanocyclohexene	90	3.87E−05	0.39	0.82
mf_21.983_57.987	Thiophene, 2‐butyltetrahydro‐	64	0.0004	0.08	0.86
mf_12.864_74.019	Dihydro‐2(3*H*)‐thiophenone	91	0.0005	0.10	0.88
mf_1.901_50.015	1‐Buten‐3‐yne	82	0.0009	2.58	0.86
mf_17.37_120.006	Disulfide, methyl 2‐propenyl	64	0.001	0.12	0.88
mf_27.998_71.086	Hexane, 3,3‐dimethyl‐	95	0.001	0.30	0.82
mf_8.379_73.011	Sulfide, allyl methyl	95	0.002	0.04	0.84
mf_18.304_57.987	Propanoic acid, 3‐(acetylthio)‐2‐methyl‐	90	0.002	0.16	0.82
mf_26.078_139.112	Cyclohexanone, 5‐methyl‐2‐(1‐methylethyl)‐, trans‐	HRAM	0.004	0.29	0.84
mf_26.48_139.112	Cyclohexanone, 5‐methyl‐2‐(1‐methylethyl)‐, trans‐	94	0.005	0.34	0.81
mf_1.988_48.003	Methanethiol	97	0.006	0.30	0.80
mf_18.836_93.07	Tricyclo[2.2.1.0(2,6)]heptane, 1,3,3‐trimethyl‐	94	0.008	0.39	0.80
mf_24.976_80.062	Fenchyl acetate	94	0.008	0.24	0.80
mf_9.357_73.011	Sulfide, allyl methyl	95	0.009	0.13	0.83
mf_19.396_125.963	Dimethyl trisulfide	68	0.013	0.33	0.80

*Note*: VOCs that were more abundant in the breath of acute leukemia patients are highlighted in orange; those that were more abundant in the breath of healthy controls are highlighted in blue.

Abbreviations: FC, fold change; HRAM, high‐resolution accurate mass; NIST, National Institute of Standards & Technology; ROC AUC, receiver operating characteristic area under the curve; TMS, trimethylsilyl.

In conclusion, this is the first report of the utility of exhaled breath VOCs analysis in patients with hematological cancers. Notably, patients with high‐grade lymphoma have an increased abundance of VOCs linked to lipid peroxidation and ferroptosis. This observation is biologically plausible, as these metabolic pathways are commonly dysregulated in cancers including lymphoma. Therefore, “breath biopsy” of these VOCs offers the potential for a rapid and noninvasive assessment of the disease burden of lymphoma patients and could be used as a biomarker of treatment response, both for existing therapies and for modulators of ferroptosis. However, this study does highlight some of the limitations of breath testing such as the confounding potential of antibacterial therapy and dietary alterations. In addition, although virtually all (96%) of the samples collected from the healthy controls were adequate for analysis, only 75% of the samples collected from blood cancers were satisfactory. Even though patients with moderate‐severe pulmonary disease were excluded, some patients were unable to complete the prolonged (~10 min) breath collection. However, this study was designed to be an untargeted analysis, which required this length of breath collection. In the future, more focused techniques (e.g., aimed at detecting lipid peroxidation products) could collect breath for a few seconds, comparable to the detection of ethanol in the breath of motorists for policing purposes, mitigating against this.

## AUTHOR CONTRIBUTIONS

**Lotte C. A. Stiekema**: Conceptualization; methodology; investigation; writing—review and editing. **Hsuan Chou**: Methodology; validation; formal analysis. **Amy Craster**: Methodology; validation; formal analysis. **Bela Wrench**: Conceptualization; writing—review and editing; resources; supervision. **Katiuscia Bianchi**: Conceptualization; writing—review and editing; supervision; resources. **Paolo Gallipoli**: Conceptualization; writing—review and editing; supervision; resources. **Jeffrey K. Davies**: Conceptualization; supervision; resources; writing—review and editing. **John G. Gribben**: Conceptualization; funding acquisition; supervision; resources; writing—review and editing. **John C. Riches**: Conceptualization; methodology; data curation; investigation; validation; formal analysis; supervision; funding acquisition; project administration; writing—review and editing; writing—original draft; resources.

## CONFLICT OF INTEREST STATEMENT

H.C. and A.C. are/have been employees of Owlstone Medical Ltd. None of the other authors has a conflict of interest regarding this study.

## ETHICS STATEMENT

The study was approved by the Bristol Research Ethics Committee, Bristol, UK (IRAS 276205). Informed consent was obtained from patients and healthy controls in all cases.

## FUNDING

This study was supported by funding from Barts Charity, London, UK (Investigation of the potential of breath biopsy in haematological malignancies [G‐001866/MRC0261]).

## Supporting information

Supporting Information.

## Data Availability

The data that support the findings of this study are available from the corresponding author upon reasonable request. All relevant information and data generated or analyzed during this study are included in this published article and its Supporting Information files.
