# Supplementary material for: Analysis of volatile organic compounds in exhaled breath of blood cancer patients identifies products of lipid peroxidation as biomarkers for lymphoma detection
Source: Hemasphere. 2025 Jul 18;9(7):e70168. doi: 10.1002/hem3.70168 (PMC12272501; doi:10.1002/hem3.70168)
Supplement: Supplementary file 1 — Supporting Information. [file HEM3-9-e70168-s001.docx]

**Supplementary Information to:**

**Analysis of volatile organic compounds in exhaled breath of blood cancer patients identifies products of lipid peroxidation as biomarkers for lymphoma detection**

**Supplementary Appendix Content:**

- Supplementary Methods
- Supplementary Tables
  - Suppl. Table 1. Inclusion and Exclusion Criteria
  - Suppl. Table 2. Clinical characteristics of patients included in the study
- Supplementary Figure 1: The ReCIVA Breath Sampler

**Supplementary Methods**

**Sample Collection**

Breath samples were collected using a ReCIVA Breath Sampler (Owlstone Medical Ltd.). During the procedure, exhaled breath was collected onto a Breath Biopsy Cartridge, which consists of four Tenax TA/Carbograph 5TD sorbent tubes (Markes International). The ReCIVA Breath Sampler monitors subjects’ breathing pattern in real time using pressure sensors. Dynamically determined gates using the real-time pressure levels trigger the sampling pumps to collect breath. The breath collect method is scaled to a typical tidal breath volume with start collection threshold corresponding to a CO_2_ level of approximately 0.6%. The first part of the breath is excluded as it predominantly consists of air from the mouth cavity and upper airway. The end of the exhalation is also excluded due to poor repeatability. Each pump pulls pressure-gated exhaled breath through two sorbent tubes, 790 mL is collected on each tube (each pair of tubes is later combined to give a single sample for TD-GC-MS analysis).

**Sample Curation (Pre-Analytical)**

Breath samples were curated using an in-house automated filtering system to score the likelihood of individual samples being of acceptable quality before initiating data analysis. Briefly, the curation tool starts by ranking the status of the shipped samples using check-in sample tags as measurements of overall sample quality. The sample curation then continues by identifying and scoring events which might have affected breath sample quality during the analytical data acquisition process. The subsequent step consists of the scoring of pressure inconsistencies which might represent potential ReCIVA Breath Sampler leak during sample collection on site. Samples deemed to have unacceptable quality at this stage were not included in data analysis.

**Sample Randomization**

The aim of sample randomization is to ensure independence between sequence and variables of interest:

- Collect timestamp
- Breath Collect Type
- Gender
- Age
- Weight

To carry out the method of randomization, categorical variables were one-hot encoded. A measure of correlation between each pair of variables was computed (Matthew’s coefficient of correlation for binary-binary correlation, Spearman's rank correlation coefficient for continuous-continuous correlation, the square root or r-squared from a linear regression for continuous-binary correlation). The matrix of pair-wise correlation was eigen decomposed to identify the most informative combinations of variables that could be used to cluster the samples. K-means clustering was then applied to cluster the samples (n cluster ≤ number of sequences). After the clustering, stratified sampling was used to select 8 samples for each sequence.

**Sample Analysis**

Samples were analyzed using the OMNI Breath Biopsy® Platform in the Breath Biopsy Laboratory (Owlstone Medical Ltd.). Samples were dry purged to remove excess water using a TD100-xr thermal desorption autosampler (Markes International) at receipt, and then stored until the final cohort had been assembled. Samples were thermally desorbed on a TD100-xr thermal desorption autosampler and chemicals in samples were chromatographically separated using a programmed method (temperature ramp) and mass spectral data were acquired using electron impact (EI) ionization and the Orbitrap high resolution accurate mass (HRAM) spectrometer (Thermo Scientific). Two sample tubes were desorbed into the Thermal Desorber cold trap for a single analysis, a sample being a combination of two sorbent tubes. A cleaning method was run in between each sample to prevent carry-over. A quality control sample (sorbent tube spiked with a known mixture of chemicals) was run in between every four subject breath samples to monitor the stability of instrumentation. A blank tube was run every four samples and after every quality control sample to monitor background. As a final step, additional column resolution checks were included to maximize the ability to extract high quality MFs. Samples passing this check were likely to have the most complete set of MFs extracted. Analytical variation associated with TD-GC/MS analysis can be assessed using relative standard deviation (RSD) of internal standards used to monitor platform performance. An average RSD < 20% is considered acceptable by Owlstone’s quality standards; *in this project a 14% RSD was observed*.

**Feature Extraction**

Raw chromatograms were imported into Compound Discoverer version 3.2 (Thermo Scientific) to perform feature extraction. Thermo Scientific proprietary algorithms were used to perform deconvolution. Deconvolution results in a list of MFs, each feature consisting of mass spectral ions with similar chromatographic characteristics. Key feature extraction parameters were:

- TIC threshold
- Ion overlap window
- Dot product
- Smoothing
- Mass tolerance
- Peak signal-to-noise
- Spectrum signal-to-noise
- Gap filling

The MFs produced by deconvolution were manually inspected by Owlstone curators to check extraction and integration consistency across the entire dataset. When a feature was not detected or not identified with satisfactory confidence in a sample, the corresponding entry in the feature table was marked as ‘data missing’.

**Imputation and normalization**

As a sparseness criterion, features that were detected in less than 80% of samples in the study’s smallest sub-group (I.e. the ALL group), were excluded. For each feature, if a missing value was the result of a signal below the limit of detection (LOD), the missing values were imputed as 80% of the minimum intensity for that feature. Missing values that were not due to below-LOD signals were imputed with the median of the corresponding feature. To remove sequence-to-sequence variation in instrument sensitivity, the untargeted features were normalized using scaling factors derived from 8 internal standards (median of the 8 internal standards in each sample).

**Molecular Feature (MF) Identification**

Tentative identifications were assigned by comparison to the National Institute of Standards & Technology (NIST) library and/or Owlstone Medical’s internal high resolution accurate mass (HRAM) library. Whereas the NIST library provides more extensive coverage of the tentative IDs representing the chemical composition of MFs, the HRAM library provides more accurate tentative identification assignment of the features based on accurate mass for the subset of candidates with a chemical standard tested at Owlstone. In addition to the tentative identifications, the MF identification also returns a score that reflects the percentage match with the library spectrum. Higher scores indicate better tentative matches against the reference libraries. However, even MFs with high scores might be identified incorrectly due to non-specificity of the reference library spectra. This is particularly true of matches made to the NIST library. Therefore, the tentative identifications are best thought of as guides to the likely general molecular formula and presence of chemical functional groups (e.g. alcohols, aldehydes, unsaturated alkanes). These tentative identifications can be confirmed by comparison to true standards for VOCs of particular interest as a component of a follow-on project.

**Univariate Analysis of VOCs**

To test whether individual VOC abundances differ between two or more groups, $Log\left( VOC \right)\sim IsCase + Covariate_{i}$ is used. The covariates that are included in the model are the demographic variables that were found to be significantly different between the two disease groups being compared. The Benjamini-Hochberg method was used to correct P-values for multiple testing with a false discovery rate of 0.1. Due to the relatively small sample size, an uncorrected P-value less than 0.05 was considered as evidence of difference or association.

**Classification Models**

*Elastic-Net Regression*

Elastic net is a regularization technique for reducing the complexity of linear models in datasets where the number of features is high compared to the sample size, as models trained on this type of data are likely to overfit. It limits overfitting by reducing the size of regression coefficients of less informative or redundant features and setting coefficients of non-informative features to zero. The regularisation in elastic net is achieved by combining both L1 and L2 constraints on the regression coefficients in a weighted fashion. The hyperparameter alpha determines the relative strength of L1 and L2 constraints. An alpha value near 1 result in L1-like behaviour, and an alpha value near 0 leads to L2-like behaviour. The L1 constraint reduces the sum of absolute values of regression coefficients, resulting in a final model where many coefficients are set to zero. In contrast, the L2 constraint reduces the sum of squared value of regression coefficients, it is more stable than L1, but results in a much less interpretable model containing many near-zero coefficients. By combining both the L1 and L2 constraints, elastic net is able to select a stable and relatively sparse model. In this study, alpha = 0.9 was chosen to achieve a relatively sparse model.

*Stability Selection*

Stability selection is a feature selection method where a high-dimensional selection algorithm such as LASSO (an L1-like elastic-net regression in this study) is combined with a random subsampling approach to identify features that are consistently selected in a large fraction of subsamples for one or more regularization strengths. Meinshausen and Bühlmann have demonstrated that this approach outperformed standard LASSO on datasets with correlated features by increasing the number of correctly selected features while reducing the number falsely selected features.

This study used a 100-split Monte Carlo cross-validation with a 1:1 train-test split as the resampling procedure. A feature was selected if it was in over 90% of models (one per split) for any regularization strength considered. The lower limit of the regularization strength was defined so the expected number of falsely selected features had a maximum of 1.

**Supplementary Table 1: Inclusion and Exclusion criteria**

| **INCLUSION CRITERIA:**  **Patient group (High-grade lymphoma; ALL)**   - Age ≥ 18 years - Diagnosis of a hematological malignancy (according to current national guidelines): ALL, high-grade lymphoma - If applicable, no current treatment for their hematological malignancy^a^   **Patient group (AML)**   - Age ≥ 18 years - Diagnosis of AML (according to current national guidelines) - Start with first induction chemotherapy within 7 days^a^   **Healthy control group**   - Age ≥ 18 years - No prior or current significant health conditions at time of screening |
| --- |
|  |
| **EXCLUSION CRITERIA:**   - Unwilling or unable to give consent - Inability to understand written and/or verbal English - Active lung disease or any clinically significant medical condition that could interfere with the conduct of the study in the opinion of the investigator^b^ - Chronic or recent (<1 month) infections and/or clinical signs of acute infection - Auto-immune diseases^c^ - Active smoking/vaping - Type I or II diabetes mellitus - Use of drugs that could interfere with the study parameters - Inability or unwillingness to comply with the protocol requirements, or deemed by investigator to be unfit for the study - Have recently tested positive for coronavirus disease-19 |

**Notes:**

^a^ The patients were all tested prior to the start of anti-neoplastic therapy except for some patients with acute leukemia who were receiving cytoreductive therapy (e.g. hydroxycarbamide) to control their blast count as required by their clinical status. In these cases, patients were still tested prior to the initiation of definitive therapy (e.g. daunorubicin + cytarabine).

^b^ This criterion aimed to exclude patients who the study investigators felt would not be able to tolerate the breath collection process. Patients with lung/pleural/bulky mediastinal involvement were included if they were able to breathe comfortably as rest and were able to tolerate the breath collection procedure.

^c^ Previous reports have demonstrated that inflammatory/auto-immune states (e.g. inflammatory bowel disease, inflammatory arthropathies) can affect the levels of breath VOCs. Therefore, patients with active autoimmune diseases or those having ongoing immunosuppressive therapies were excluded. Patients who were asymptomatic and were not taking immunosuppressants were included (e.g. mild asthma).

**Supplementary Table 2: Clinical characteristics of patients included in the study**

| **High-grade Lymphoma** | | | | | |
| --- | --- | --- | --- | --- | --- |
| **ID** | **Age** | **Sex** | **Blood Cancer (Stage/Pathology)** | **Other Medical History** | **Antibacterial** |
| BI1002 | 71 | M | New BL (Stage IV/MYC rearranged) | Nil |  |
| BI1003 | 53 | M | R/R Blastoid MCL (Stage IV) | Nil | Co-amoxiclav |
| BI1018 | 73 | M | New DLBCL (Stage IIS/ MYC and BCL2 rearranged) | Previous MI |  |
| BI1022 | 70 | M | New Blastoid MCL (Stage IVS) | Hypothyroidism |  |
| BI1029 | 47 | M | New DLBCL (PTLD) (Stage IVS/GCC; EBV-; not rearranged) | Previous renal Tx |  |
| BI1041 | 68 | F | New MCL (Stage IV) | Osteoarthritis |  |
| BI1042 | 59 | M | Newly tFL -> DLBCL (Stage IV) | Nil |  |
| BI1044 | 58 | M | New DLBCL (Stage II Bulky/ABC; EBV+; not rearranged) | HIV+ |  |
| BI1049 | 66 | M | New DLBCL (Stage IV/ABC; DE, not rearranged) | Nil |  |
| BI1051 | 46 | M | New DLBCL (Stage IV/ABC; DE, not rearranged) | Nil | Co-amoxiclav |
| BI1054 | 69 | M | New TCR-BCL (Stage IV) | Previous hepatitis B |  |
| BI1055 | 55 | M | New DLBCL (Stage IV/DE, MYC rearranged) | HTN, HCA, Crohn’s |  |
| BI1060 | 58 | M | New tLPL -> DLBCL (Stage IV/ ABC, MYC rearranged) | Previous TB and PCP |  |
| BI1064 | 32 | M | R/R DLBCL (Stage IV) | Nil |  |
| BI1067 | 56 | M | New MCL (Stage IV) | Nil |  |
| BI1079 | 44 | M | New DLBCL (Stage IV/ABC; EBV+) | Previous necrotising fasciitis R thigh |  |
|  | | | | | |
| **Acute Myeloid Leukemia** | | | | | |
| **ID** | **Age** | **Sex** | **Diagnosis (Genetics)** | **Other Medical History** | **Antibacterial** |
| BI1006 | 68 | F | New AML  (+8q; NPM1/FLT3 wt) | Nil | Ciprofloxacin |
| BI1011 | 61 | F | New AML  (RUNX1 mut.; NPM1/FLT3 wt) | Nil |  |
| BI1013 | 23 | M | New AML (t15;17 - not PML-RARA; NPM1 wt/FLT3 mut.) | Nil | Tazocin |
| BI1014 | 56 | F | New AML (Complex karyotype, TP53 mut. NPM1/FLT3 wt) | Nil |  |
| BI1020 | 62 | M | New AML | HTN, BPH | Ciprofloxacin |
| BI1033 | 58 | M | New AML  (FLT3-ITD+, NMP1+) | Nil | Ciprofloxacin |
| BI1035 | 72 | M | CMML -> AML  (5q-, 8q+; NMP1/FLT3 wt) | HTN, AF, HCA, BPH |  |
| BI1036 | 67 | M | New AML (Tri 10; CEBPA mut. FLT3 and NMP1 wt) | Renal stones, lumbar surgery |  |
| BI1045 | 52 | F | New AML  (11q+, 8q+; NMP1wt/FLT3 TKD+) | Previous obesity, Abdominoplasty | Ciprofloxacin |
| BI1050 | 79 | F | New AML (Mono 7; RUNX1 mut.; NPM1/FLT3 wt) | HTN |  |
| BI1052 | 70 | F | New AML (normal cytogs. FLT3 ITD/NPM1 mut.) | HCA | Co-amoxiclav, Doxycycline |
| BI1053 | 25 | F | New AML (+8, +22; RUNX1/IDH1 mut. NMP1/FLT3 wt) | Nil | Tazocin |
| BI1058 | 70 | F | New AML  (CEBPA mut.; FLT3 and NPM1 wt) | OSA, IBS, Psoriasis, RA |  |
| BI1062 | 77 | F | R/R AML  (9q-; FLT3 ITD/NPM1 mut.) | Previous breast cancer, mild asthma | Tazocin |
| BI1063 | 67 | F | New AML (7q-; RUNX1/IDH1/DNMT3A mut.; NMP1 wt./FLT3 ITD) | Nil |  |
| BI1065 | 41 | F | New AML (normal cytogs; GATA2/CEBPA mut.) | Nil |  |
| BI1069 | 43 | F | New AML  (2q-, NPM1/FLT3 wt) | Previous hepatitis B | Tazocin, Amikacin |
| BI1081 | 48 | M | New AML (normal cytogs; IDH1 mut.; NPM1 mut./FLT3 wt_ | Nil |  |
| BI1082 | 67 | M | New AML (RUNX1 mut. NPM1/FLT3 wt) | Mild asthma, HCA, Angina, AF |  |
| BI1083 | 58 | F | New AML (normal cytogs, NRAS mut.; NPM1 mut./FLT3 TKD) | Renal stones, Mild COPD, Angina | Ciprofloxacin, Vancomycin |
|  | | | | | |
| **Acute Lymphoblastic Leukemia** | | | | | |
| **ID** | **Age** | **Sex** | **Diagnosis (Genetics)** | **Other Medical History** | **Antibacterial** |
| BI1001 | 29 | M | R/R Pro T-ALL (1p-; 5q35 TLX3 rearranged; NUP214-ABL1 fusion) | Nil | Penicillin |
| BI1016 | 52 | F | New Pre B-ALL (t(9;22); BCR/ABL1 fusion) | Hypothyroidism |  |
| BI1017 | 53 | M | New B-ALL (inv 3, t(9;22), +6; BCR/ABL1 fusion) | Nil |  |
| BI1019 | 74 | M | R/R B-ALL (t(9;22); BCR/ABL1 fusion) | Nil |  |
| BI1037 | 32 | M | R/R Cortical T-ALL (XXY, t(11;14) +20) | Nil | Co-trimoxazole |
| BI1057 | 63 | M | New B-ALL (49 XY, T(2;5), Inv 7, +8, +17) | HTN, HIV |  |
| BI1059 | 64 | F | New common B-ALL (Hypodiploid: 38-41XX) | Sickle cell trait, Previous laparotomy | Meropenem |
| BI1061 | 54 | F | New common B-ALL (t(9;22); BCR/ABL1 fusion) | Previous Ovarian Cyst | Ciprofloxacin, Vancomycin |
| BI1066 | 26 | F | New T-ALL (normal cytogs; NOTCH1 mut.; PTEN mut.) | Mild asthma | Co-trimoxazole |
| BI1071 | 30 | M | New mixed phenotype AL; predominantly T-ALL (+1p; +15p; NRAS mut.) | Nil |  |

**Abbreviations:** AF: atrial fibrillation; ALL: acute lymphoblastic leukemia; AML: acute myeloid leukemia; BL: Burkitt lymphoma; BPH: benign prostatic hypertrophy; COPD: chronic obstructive pulmonary disease; DLBCL: diffuse large B-cell lymphoma; F: female; HCA: hypercholesterolemia; HIV: human immunodeficiency virus; HTN: hypertension; IBS: irritable bowel syndrome, M: male; MCL: mantle cell lymphoma; MI: myocardial infarction; OSA: obstructive sleep apnoea; PCP: pneumocystis pneumonia; RA: rheumatoid arthritis; R/R: relapse/Refractory; TB: tuberculosis; TCR-BCL: T-cell rich B-cell lymphoma; tFL: transformed follicular lymphoma; tLPL: transformed lymphoplasmacytic lymphoma; Tx: transplant


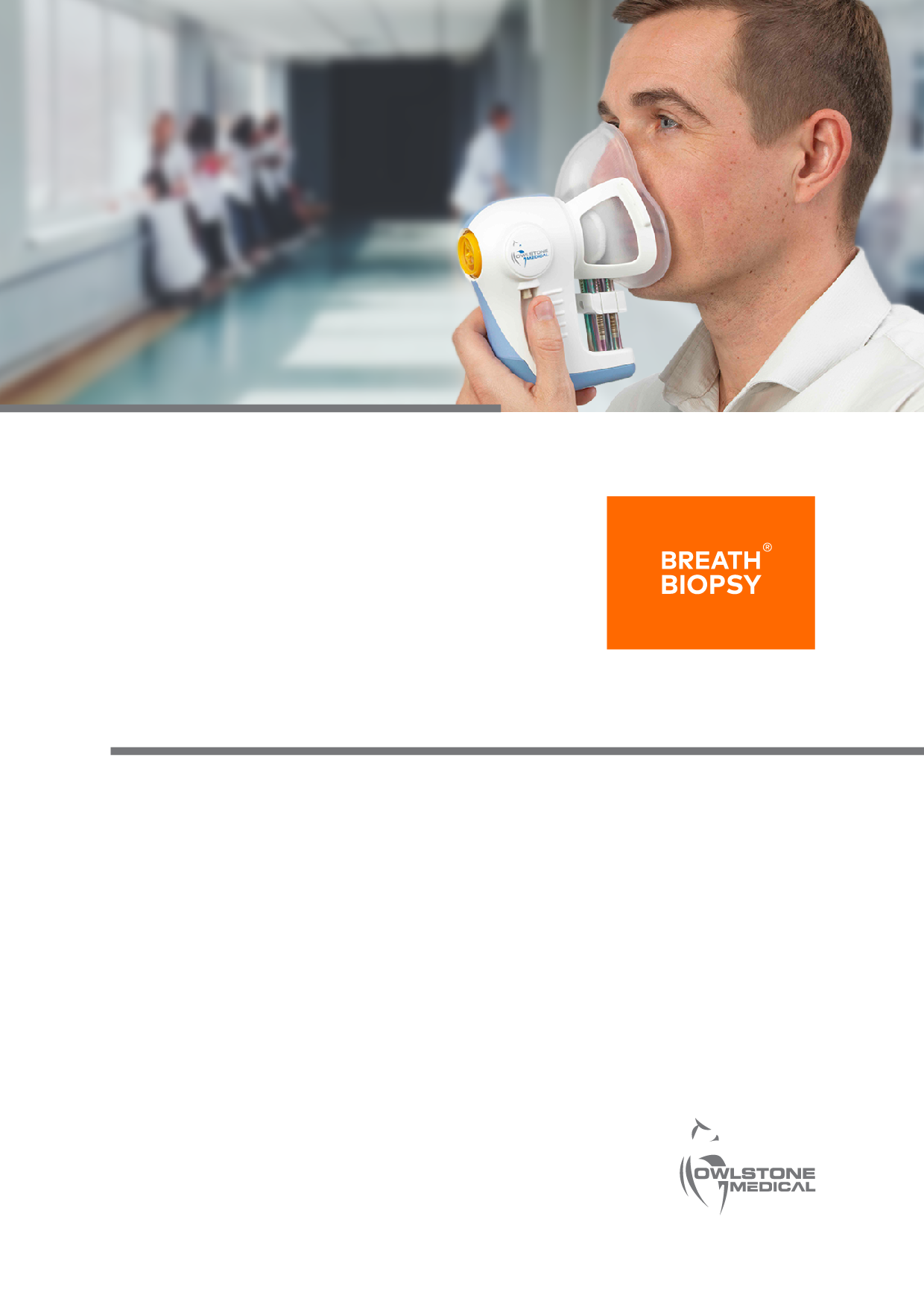


**Supplementary Figure 1. The ReCIVA Breath Sampler**
